# Diagnostic Efficiency of Artificial Intelligence Integrated Intraoral Mobile Photographs in Identification of Oral Potentially Malignant Disorders: An Umbrella Review

**DOI:** 10.1155/ijod/6654212

**Published:** 2026-10-01

**Authors:** P. D. Madan Kumar, C. Lavanya, Sasidharan Sivakumar, K. Ranganathan, M. Pavithra, R. Palanivel Pandian, B. R. Avinash

**Affiliations:** ^1^ Department of Public Health Dentistry Ragas Dental College and Hospital Chennai India; ^2^ Department of Oral Pathology and Microbiology Ragas Dental College and Hospital Chennai India; ^3^ Discovery Research Division Indian Council of Medical Research ICMR Headquarters Ansari Nagar New Delhi India icmr.nic.in; ^4^ Department of Oral and Maxillofacial Pathology Ragas Dental College and Hospital Chennai India; ^5^ Department of Public Health Dentistry Best Dental Sciences College and Hospital Madurai 625104 Tamil Nadu India; ^6^ Department of Public Health Dentistry Manipal College of Dental Sciences Mangalore Manipal Academy of Higher Education Manipal 576104 India manipal.edu

**Keywords:** artificial intelligence, diagnostic accuracy, mobile photography, noninvasive screening, oral potentially malignant disorders (OPMDs), oral squamous cell carcinoma (OSCC), resource-limited settings

## Abstract

**Background:**

Oral potentially malignant disorders (OPMDs) are precursors to oral squamous cell carcinoma (OSCC), a malignancy with high morbidity and mortality due to late diagnosis. Conventional diagnostic methods, though accurate, are invasive and often inaccessible in resource‐limited areas. Intraoral mobile photographs integrated with artificial intelligence (AI) as a screening tool may offer a noninvasive, cost‐effective, and accessible alternative for early detection of OPMDs. The study aimed to evaluate the diagnostic accuracy of mobile photographic models in identifying OPMDs.

**Settings and Design:**

An umbrella review synthesizing evidence from previously published systematic reviews/meta‐analyses was carried out following Preferred Reporting Items for Systematic Reviews and Meta‐Analyses (PRISMA) guidelines.

**Methods and Material:**

Databases including PubMed, Scopus, Embase, Web of Science, and through supplementary search engines like Google Scholar were searched. Inclusion criteria encompassed studies analyzing the diagnostic performance of mobile photographic models for OPMDs. Sensitivity, specificity, and applicability were examined.

**Results:**

The pooled sensitivity was 90% (95% CI: 88.8%–92.6%) and specificity was 89% (95% CI: 86%–92%). Minimal heterogeneity in sensitivity (*I* ^2^ = 34.01%) and higher variability in specificity (*I* ^2^ = 93.34%) were observed. Funnel plots confirmed no publication bias.

**Conclusions:**

Intraoral mobile photographs were effective in noninvasive OPMD detection, particularly in low‐resource settings. Further research should aim to refine algorithms, standardize imaging, and validate performance in real‐world scenarios.

## 1. Introduction

Oral squamous cell carcinoma (OSCC) constitutes a significant global health burden, with disproportionately high incidence and mortality rates in low‐ and middle‐income countries (LMICs) [1]. The primary challenge lies in the late‐stage presentation of the disease, which substantially limits therapeutic success and adversely impacts survival outcomes. Early detection of oral potentially malignant disorders (OPMDs), such as leukoplakia, erythroplakia, and oral submucous fibrosis, is therefore essential to intercept malignant progression and reduce the disease burden [2].

Numerous studies have established the diagnostic utility of conventional approaches, including biopsy and histopathological examination [3]. While these methods remain the gold standard, they are invasive, costly, and often inaccessible in primary healthcare settings. Consequently, there is a pressing need for alternative, scalable, and noninvasive diagnostic strategies that can support large‐scale screening in resource‐constrained environments [4].

Recent advancements in artificial intelligence (AI), particularly the development of applications that increase disease prediction by uploading photographs to pocket‐friendly digital devices with updated high‐quality cameras, have demonstrated promising results in the field of medical imaging [5]. Mobile oral cavity photography integrated with AI offers a low‐cost, noninvasive, and portable solution for the detection and surveillance of OPMDs. These tools have the potential to overcome existing barriers to early diagnosis, especially in underserved populations [6].

However, despite their emerging potential, the current literature lacks comprehensive synthesis regarding the diagnostic performance, consistency, and methodological quality of such AI‐driven approaches [7]. This umbrella review aims to address this gap by evaluating the diagnostic accuracy of intraoral mobile photographs coupled with AI in detecting OPMDs, explaining how and why such tools could be instrumental in transforming community‐level screening strategies, and providing an evidence‐based rationale for their broader clinical integration.

Although several systematic reviews and meta‐analyses have evaluated the application of AI for the detection and classification of oral OPMDs and OSCC, the existing evidence remains fragmented. Most reviews have focused on specific AI algorithms, individual imaging modalities, or selected clinical outcomes, resulting in considerable variation in study populations, datasets, reference standards, performance metrics, and methodological quality. Furthermore, the rapid evolution of AI technologies has led to the publication of multiple overlapping systematic reviews with differing conclusions, making it challenging for clinicians, researchers, and policymakers to identify the most reliable evidence. To date, no comprehensive umbrella review has systematically synthesized, critically appraised, and compared the findings of these existing systematic reviews and meta‐analyses while simultaneously evaluating their methodological quality, risk of bias, and certainty of evidence. Therefore, the present umbrella review was undertaken to provide a higher level of evidence by integrating and critically evaluating the current body of synthesized literature, identifying areas of consensus and inconsistency, highlighting methodological limitations, and outlining priorities for future research and clinical translation of AI‐assisted diagnostic technologies in oral cancer screening.

This umbrella review critically evaluates the potential clinical application of AI‐assisted oral mobile photographs in enhancing diagnostic performance, supporting clinical decision‐making, and facilitating the early detection of OPMDs.

## 2. Materials and Methods

### 2.1. Protocol and Registration

This systematic review was designed in accordance with the Preferred Reporting Items for Systematic Reviews and Meta‐Analyses (PRISMA) guidelines. The study protocol was prospectively registered in the International Prospective Register of Systematic Reviews (PROSPERO) under the Registration ID CRD42024599720. The complete electronic search strategy, particularly the database and the search terms, are in accordance with Table 1.

**Table 1:** Search strategy for the study.

Database	Numbers	Search strategy
Medline (PubMed)	15	(“mouth”[MeSH Terms] OR “mouth”[All Fields] OR “oral”[All Fields]) AND (“precancerous conditions”[MeSH Terms] OR (“PRECANCEROUS”[All Fields] AND “conditions”[All Fields]) OR “precancerous conditions”[All Fields] OR “premalignant”[All Fields] OR “premalign”[All Fields] OR “premalignancies”[All Fields] OR “premalignancy”[All Fields]) AND “PRECANCEROUS”[All Fields] AND (“mouth neoplasms”[MeSH Terms] OR (“mouth”[All Fields] AND “neoplasms”[All Fields]) OR “mouth neoplasms”[All Fields] OR (“oral”[All Fields] AND “cancer”[All Fields]) OR “oral cancer”[All Fields]) AND (“diagnosis”[MeSH Terms] OR “diagnosis”[All Fields] OR “diagnostic”[All Fields] OR “diagnostical”[All Fields] OR “diagnostically”[All Fields] OR “diagnostics”[All Fields]) AND (“artificial intelligence”[MeSH Terms] OR (“artificial”[All Fields] AND “intelligence”[All Fields]) OR “artificial intelligence”[All Fields])
Web Of Science (WoS)	53	((((ALL = (“oral premalignant lesion”)) OR ALL = (“oral premalignant”)) OR ALL = (“oral premalignant condition”)) OR ALL = (“oral lesion”)) OR ALL = (“oral lesions”) AND (((((ALL = (“intraoral photography”)) OR ALL = (“intraoral photographs”)) OR ALL = (“oral images”)) OR ALL = (“photographic evaluation”)) OR ALL = (photograph)) OR ALL = (photographs)
Scopus	639	((ALL (“intraoral lesions”) OR ALL (“intraoral lesion”) OR ALL (“premalignant lesions”) OR ALL (“premalignant lesion”) OR ALL (“premalignant condition”) OR ALL (“precancerous lesions”) OR ALL (“precancerous conditions”) OR ALL (“precancerous lesion”) OR ALL (precancer))) AND ((ALL (“intraoral photography”) OR ALL (“intraoral photograph”) OR ALL (photographs) OR ALL (photography) OR ALL (photograph)))
Embase	68	((“precancer”/exp OR “praecancer” OR “pre cancer” OR “pre‐cancerous condition” OR “pre‐cancerous lesion” OR “pre‐cancerous state” OR “pre‐malignancies” OR “pre‐malignancy” OR “pre‐malignant condition” OR ”pre‐malignant lesion” OR “pre‐malignant state” OR “pre‐neoplastic condition” OR “precancer” OR “precanceroses” OR “precancerosis” OR “precancerous conditions” OR “precancerous lesion” OR “precancerous state” OR “premalignancies” OR “premalignancy” OR “premalignant condition” OR “premalignant lesion” OR “premalignant state” OR “preneoplastic condition” OR “preneoplastic state”) OR (“mouth lesion”/exp OR “intraoral lesion” OR “mouth lesion” OR “oral lesion” OR “oral mucosa wound”)) AND (“tooth radiography”/exp OR “intraoral photography” OR “photography, intraoral”)
Google Scholar	23	(“oral premalignancy” OR “oral cancer” OR “oral precancer” OR “OPMD precancerous”) AND (“dental photography” OR “intraoral photography”)

### 2.2. Study Design and Period

This study was a protocol‐driven umbrella review designed to assess the diagnostic precision of intraoral mobile photographs predicted by AI in identifying OPMDs from 01/01/2010 to 30/06/2026. The methodological framework was established to ensure transparency, reproducibility, and consistency with reporting standards for diagnostic accuracy studies.

### 2.3. Ethical Considerations

Ethical clearance was not required, and the reason for exemption was that our study involved secondary analysis of data from previously published literature, which were all available in the public domain. It was assumed that all included studies had adhered to the ethical principles outlined in the Declaration of Helsinki (as revised in 2000) and received appropriate institutional or regional ethical clearance.

### 2.4. Eligibility Criteria

The inclusion criteria were defined in advance and included systematic reviews that

• Involved human participants with clinically or histologically diagnosed OPMDs,
• Employed intraoral mobile photographs for lesion detection,
• Reported diagnostic performance metrics such as sensitivity, specificity, or accuracy and
• Compared AI‐driven oral mobile photographic images with conventional diagnostic standards (e.g., histopathology),
• Cross‐sectional study designs.

Exclusion criteria included the following:

• Studies lacking quantitative diagnostic accuracy outcomes,
• Articles not published in English,
• Studies that did not utilize intraoral mobile photographs,
• Research with incomplete methodology or lacking adequate validation of AI systems.

Reviewer calibration/pilot screening

Before commencing the formal screening process, the reviewers conducted a pilot screening exercise using a representative sample of retrieved records to ensure a consistent interpretation of the eligibility criteria. Any discrepancies identified during the pilot phase were discussed and resolved, and the screening criteria were refined at junctures where it was necessary before full‐text screening commenced.

Resolution of disagreements

Study selection, data extraction, and methodological quality assessment were performed independently by two reviewers. Any disagreements were resolved through discussion and consensus. When consensus could not be reached, a third reviewer was consulted to make the final decision.

As this study was conducted as an umbrella review, the potential overlap of primary studies across the included systematic reviews was considered during the evidence synthesis. The included reviews were examined to identify substantial duplication of evidence; however, a formal qualitative assessment of overlap was performed.

### 2.5. Data Extraction and Quality Assessment

Prior to initiating the review, a structured data extraction form was developed to capture relevant study parameters, including sample characteristics, AI model descriptions, reference standards, and diagnostic accuracy outcomes. Two reviewers were designated to independently extract data and resolve discrepancies through discussion. Risk of bias and methodological quality were preplanned to be evaluated using criteria based on study design, participant selection, reference standards, and flow/timing, consistent with AMSTAR‐2, PRIOR, and JBI.

## 3. Results

### 3.1. Study Selection

A total of 798 records were identified initially (Table 1). A manual check of the titles made us exclude 57 similar titles. Out of the total 741 studies, 693 did not meet the desired study design in PICOS criteria and were thus excluded. Further, on following title and abstract screening with predefined inclusion criteria, 42 articles were subtracted from 48, which also did not fulfill one of the components of PICOS, resulting in the analysis of 6 articles. The PRISMA flow diagram (Figure 1) outlines the study selection process. These studies collectively evaluated the diagnostic performance of intraoral mobile photographs driven by AI in detecting OPMDs and other clinically relevant oral mucosal lesions.

**Figure 1:**
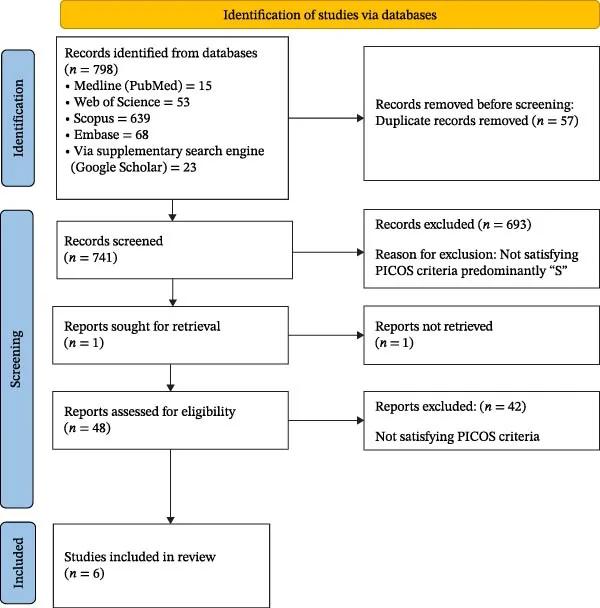
PRISMA flow chart on study selection process.

The characteristics of the included studies are summarized in Table 2. The selected studies varied in terms of geographical location, population demographics, sample sizes (ranging from 120 to 500 participants), mobile photography utilized, and reference standards. Despite methodological heterogeneity, all studies employed intraoral photographs and AI for image classification and lesion detection, providing a substantive basis for pooled analysis.

**Table 2:** Study characteristics and diagnostic performance.

Serial number	Study	Sample size	AI model used	Sensitivity (%)	Specificity (%)	Key findings
1	Kim et al. [7]	350	CNN	91.2 (86.8–94.2)	87.7 (83.2–91.3)	Demonstrated high diagnostic reliability in classifying OPMDs, indicating strong potential for early detection and clinical integration.
2	Uhrin et al. [8]	400	CNN	92.3 (83.5–96.6)	98.7 (94.7–99.7)	Showcased exceptional accuracy in telediagnosis, highlighting its viability for remote screening and diagnosis of oral lesions.
3	Ferro et al. [9]	300	CNN	91.1 (84.8–95.0)	NIL	Effectively utilized machine learning for robust lesion detection, though specificity data was not provided, necessitating further validation.
4	Li et al. [10]	200	CNN	89.9 (86.6–92.5)	89.2 (85.1–92.2)	Demonstrated consistent diagnostic performance across diverse populations, supporting its applicability in varied clinical settings.
5	Rokhshad et al. [11]	310	CNN	91 (82–96)	94 (78–98)	Proven effective in distinguishing precancerous and malignant lesions, reinforcing its role in early intervention strategies.
6	Sahoo et al.[12]	296	CNN	87 (85–89)	81 (78–84)	AI demonstrated excellent diagnostic accuracy for detecting oral potentially malignant disorders and oral cancer. The review concluded that AI has strong potential as a reliable adjunct for early detection, particularly in resource‐limited settings.

### 3.2. Forest Plot Analysis

The forest plots (Figure 2) for sensitivity and specificity reveal strong effect size estimates. For sensitivity, the estimated intercept is 0.907 (SE = 0.010, *z* = 94.045, and *p*  < 0.001) with a 95% confidence interval from 0.888 to 0.926, signifying high sensitivity and precision in diagnosing true positives. For specificity, the intercept is estimated at 0.93.34 (SE = 0.025, *z* = 37.210, and *p*  < 0.001) with a 95% confidence interval from 0.875 to 0.969, indicating acceptable specificity and confidence in excluding false positives. These high sensitivity and specificity estimates, coupled with significant *z*‐values and narrow confidence intervals, reinforce the diagnostic reliability of mobile photography.

**Figure 2:**
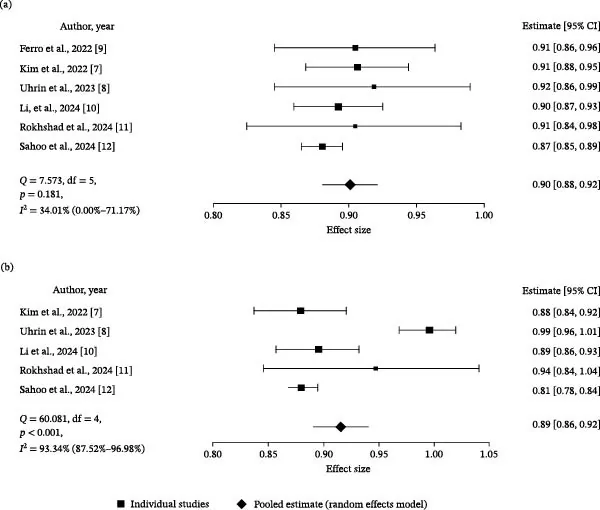
Forest plot analysis of the included studies. (a) Sensitivity and (b) specificity.

### 3.3. Funnel Plot and Publication Bias

Funnel plot analysis (Figure 3) demonstrated visual symmetry and does not indicate a potential publication bias. The Egger’s test does not support the presence of funnel plot asymmetry (intercept: 1.24, 95% CI:−0.27 to 2.74, *t*: 1.611, and *p*‐value: 0.182). This finding implies that both high‐ and low‐effect‐size studies were well‐represented in the analysis, providing a credible and comprehensive representation of the available evidence.

**Figure 3:**
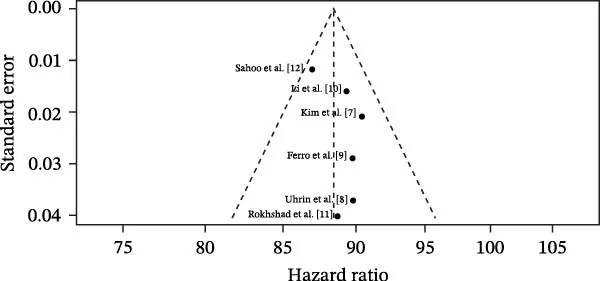
Funnel plot analysis for publication bias of the included studies.

### 3.4. Heterogeneity

Quantitative heterogeneity analysis revealed variation in sensitivity outcomes (*I* ^2^ = 34.01%), suggesting acceptable performance across populations and protocols. In contrast, specificity showed substantial heterogeneity (*I* ^2^ = 93.34%), possibly due to differences in imaging devices, lesion characteristics, or algorithmic thresholds.

### 3.5. Risk of Bias

Quality assessment indicated a low overall risk of bias in most studies, particularly in participant selection and examiner blinding. These findings underscore the need for greater standardization and transparency in future reporting.

### 3.6. Model Robustness

Intraoral mobile photographs predicted by AI consistently demonstrated strong diagnostic capability across the included studies. Higher pooled sensitivity and specificity with limited heterogeneity support their reliability in detecting true positives. Although the specificity was more variable, it remained within clinically acceptable thresholds. These findings affirm the potential of mobile device‐captured intraoral photography predicted by AI in imaging as a noninvasive, scalable diagnostic tool for early OPMD screening, especially in settings with limited access to conventional diagnostics.

## 4. Discussion

### 4.1. Summary of Key Findings

This systematic review and meta‐analysis rigorously evaluated the diagnostic utility of intraoral mobile photographs integrated with AI in the detection of OPMDs [8]. The aggregated findings underscore the strong diagnostic performance of these AI‐driven tools, evidenced by a pooled sensitivity of 90.7% and a specificity of 93.34%. These results affirm the models’ capability to accurately identify true‐positive and true‐negative cases of OPMDs, suggesting a clinically reliable, noninvasive alternative to conventional diagnostic modalities. The high sensitivity minimizes the risk of missed diagnoses—critical for conditions with malignant potential—while the high specificity mitigates the burden of unnecessary biopsies and referrals [9].

Secondary analyses revealed acceptable heterogeneity in sensitivity (*I* ^2^ = 34.01%), indicating consistent true‐positive detection across studies. Conversely, substantial heterogeneity in specificity (*I* ^2^ = 93.34%) suggests variability in false‐positive classification, warranting further investigation into underlying methodological differences, including imaging conditions, AI training protocols, and lesion heterogeneity [11]. These insights are particularly valuable in light of the study’s pre‐established hypothesis that intraoral mobile photography integrated with AI for predictive analysis facilitates diagnostics, which are both accurate and generalizable tools suitable for mass screening in resource‐limited settings [12].

The methodological quality of the included reviews was evaluated using AMSTAR‐2, which demonstrated variability in methodological rigor. Common limitations included incomplete reporting of protocol registration, inadequate assessment of publication bias, inconsistent consideration of the risk of bias in primary studies, and differences in evidence‐synthesis methods. Furthermore, although the potential overlap of primary studies across the included systematic reviews was considered during evidence synthesis, assessment using the corrected covered area (CCA) was performed, and therefore some duplication of primary evidence was excluded.

Despite the encouraging diagnostic accuracy reported across the included reviews, several challenges limit the immediate translation of AI‐assisted screening into routine clinical practice. Most primary studies were retrospective, conducted in single‐center settings, and relied on relatively small or institution‐specific datasets with limited external validation. Differences in image acquisition protocols, annotation methods, and patient populations further restrict the generalizability of the available evidence. Additional barriers, including the need for standardized imaging protocols, prospective multicenter validation, regulatory approval, integration into clinical workflows, and improved model transparency, should also be addressed before widespread implementation.

Overall, the current evidence supports the potential of AI‐assisted clinical imaging as an adjunct to the early detection of OPMDs and OSCC rather than a replacement for clinical examination and histopathological diagnosis.

### 4.2. Strengths and Limitations of the Study

A major strength of this study lies in its methodological rigor. The protocol was prospectively registered with PROSPERO (CRD42024599720), and the review process adhered to PRISMA guidelines, ensuring transparency, reproducibility, and minimized bias. Data extraction and risk of bias assessment were performed independently by two reviewers using a standardized approach‐aligned framework. The inclusion of diverse studies across geographic, ethnic, and clinical settings enhanced the external validity and applicability of the findings [13].

However, several limitations merit discussion. First, notable variability was observed in the imaging equipment (ranging from clinical‐grade intraoral cameras to consumer smartphones), and lesion types were evaluated. These inconsistencies may have contributed to the heterogeneity in specificity and restricted the generalizability of results [14, 15]. Second, the lack of standardization in model training datasets and diagnostic thresholds across studies limited the potential for meta‐regression or subgroup analysis [16]. Additionally, most studies employed retrospective or convenience sampling methods, which may introduce selection bias [17]. The exclusion of non‐English publications could also have limited access to potentially relevant data, contributing to a narrower evidence base.

### 4.3. Interpretation and Implications in the Context of Totality of Evidence

This review adds significant value to the evolving literature on AI‐based oral diagnostics. While prior systematic reviews have broadly examined AI applications in dentistry, this study uniquely focuses on intraoral mobile photography to facilitate it as a reliable screening tool for OPMDs—a subfield that holds immense potential for transforming population‐level screening strategies [18, 19].

The findings highlight an essential shift in the diagnostic paradigms. Traditionally, oral mucosal lesions were evaluated by trained clinicians using visual‐tactile examination, often followed by biopsy [20]. However, such approaches are resource‐dependent and impractical for large‐scale implementation, particularly in LMICs. The integration of AI‐powered intraoral mobile photography‐based diagnostics democratizes early detection by leveraging widely available smartphone technology, empowering primary healthcare providers and community health workers to conduct preliminary screenings [21]. The capacity for automated lesion classification enables timely triage, potentially improving early intervention and reducing oral cancer incidence [22].

These findings also support ongoing efforts in public health to decentralize specialist‐dependent workflows. By reducing diagnostic delays and enhancing accessibility, AI‐powered tools may bridge existing gaps in the cancer screening infrastructure [23]. Their deployment in rural, underserved, or mobile clinical settings could significantly relieve the burden on tertiary care centers and support the goals of national oral cancer prevention programs [24]. Moreover, the immediacy of AI‐based interpretation could enhance telemedicine frameworks, allowing remote consultations with specialists for second opinions or treatment planning [25].

### 4.4. Controversies Raised by the Study

Despite the promising outcomes, this review also surfaces several unresolved challenges and debates within the AI in healthcare domain. One primary concern is the generalizability of the intraoral images captured on the smartphones available in varied brands and prices in the market depending upon different levels of high‐definition capturing features and region‐specific or demographically homogenous datasets [26]. Variations in lesion morphology are influenced by genetic, ethnic, environmental, and behavioral factors such as tobacco usage like betel, etc. and alcohol may result in misclassification when models are applied to populations not represented in the training data [27].

AI has learned patterns from numerous images, but the exact combination of features influencing its decision is difficult to interpret. Another ongoing controversy relates to the “black box” nature of deep learning algorithms. While AI can outperform manual diagnostic methods in terms of speed and accuracy, the lack of transparency in decision‐making processes complicates clinical validation and acceptance [28]. This issue is compounded by inconsistent reporting of AI model parameters, hyperparameters, and training‐validation processes, which hinders reproducibility and peer scrutiny. Moreover, the use of diverse and often nonstandardized imaging protocols across studies further exacerbates the variability in performance metrics [29].

Finally, ethical considerations regarding data privacy, informed consent for image capture, and regulatory oversight of AI‐based diagnostics remain contentious. These aspects require robust governance structures before widespread clinical deployment can be ethically and legally justified.

### 4.5. Future Research Directions

Future research should prioritize the development and validation of standardized, open‐access, multicenter datasets representing a broad range of lesion types, ethnicities, and imaging conditions. Prospective cohort studies and randomized diagnostic trials are needed to evaluate real‐world effectiveness, particularly in primary care and community settings. Comparative studies assessing AI‐assisted diagnostics against gold‐standard histopathology will further elucidate their clinical validity. Future research should prioritize robust external validation and implementation research to strengthen the evidence base and facilitate safe and effective clinical adoption.

Advancing model interpretability through explainable AI techniques will also be crucial in fostering clinician trust and facilitating regulatory approval. Incorporating multimodal data such as patient history, risk factors, and adjunctive clinical features into AI models could enhance the diagnostic accuracy and provide a more holistic assessment of malignancy risk. Furthermore, implementation of science frameworks should be employed to explore the logistical feasibility, user acceptability, and cost‐effectiveness of deploying intraoral mobile photography integrated with AI for prediction as a screening tool within existing health systems.

From a health policy perspective, guidelines and ethical frameworks for AI deployment must be developed in tandem with technological advances to safeguard patient rights while promoting innovation. Interdisciplinary collaborations between computer scientists, clinicians, and public health professionals will be vital to translating these findings into sustainable, equitable, and impactful screening programs.

## Author Contributions

The authors have nothing to report.

## Funding

This study was done as part of the project funded by the Indian Council of Medical Research (Project ID IIRP‐2023‐1049).

## Disclosure

All authors have read and approved the final version of the manuscript. P. D. Madan Kumar had full access to all of the data in this study and takes complete responsibility for the integrity of the data and the accuracy of the data analysis. Contribution from every author is in accordance with the CRediT Taxonomy. The study was presented in the 29th National IAPHD Conference under the scientific paper delegate category by P. D. Madan Kumar.

## Ethics Statement

This study is in accordance with the ICMJE recommendations as well.

## Conflicts of Interest

The authors declare no conflicts of interest.

## Data Availability

The authors confirm that the data supporting the findings of this study are available upon reasonable request.
